# Development of a New Submaximal Walk Test to Predict Maximal Oxygen Consumption in Healthy Adults

**DOI:** 10.3390/s21175726

**Published:** 2021-08-25

**Authors:** Hyuk In Yang, Wonhee Cho, Dong Hoon Lee, Sang-Hoon Suh, Justin Y. Jeon

**Affiliations:** 1 Exercise Medicine Center for Diabetes and Cancer Patients, ICONS, Yonsei University, Seoul 03722, Korea; hyukin.yang@yonsei.ac.kr (H.I.Y.); wcho02@syr.edu (W.C.); 2 Department of Sport Industry Studies, Yonsei University, Seoul 03722, Korea; 3 Department of Exercise Science, David B. Falk College of Sport and Human Dynamics, Syracuse University, Syracuse, NY 13210, USA; 4 Department of Nutrition, Harvard T.H. Chan School of Public Health, Boston, MA 02115, USA; dol677@mail.harvard.edu; 5 Department of Physical Education, Yonsei University, Seoul 03722, Korea; ssh@yonsei.ac.kr; 6 Cancer Prevention Center, Yonsei Cancer Center, Yonsei University College of Medicine, Yonsei University, Seoul 03722, Korea

**Keywords:** submaximal exercise protocol, maximal oxygen consumption, cardiopulmonary fitness, heart rate, walk test

## Abstract

The aim of the study was to develop a simple submaximal walk test protocol and equation using heart rate (HR) response variables to predict maximal oxygen consumption (VO_2_max). A total of 60 healthy adults were recruited to test the validity of 3 min walk tests (3MWT). VO_2_max and HR responses during the 3MWTs were measured. Multiple regression analysis was used to develop prediction equations. As a result, HR response variables including resting HR and HR during walking and recovery at two different cadences were significantly correlated with VO_2_max. The equations developed using multiple regression analyses were able to predict VO_2_max values (r = 0.75–0.84; r^2^ = 0.57–0.70; standard error of estimate (SEE) = 4.80–5.25 mL/kg/min). The equation that predicted VO_2_max the best was at the cadence of 120 steps per minute, which included sex; age; height; weight; body mass index; resting HR; HR at 1 min, 2 min and 3 min; HR recovery at 1 min and 2 min; and other HR variables calculated based on these measured HR variables (r = 0.84; r^2^ = 0.70; SEE = 4.80 mL/kg/min). In conclusion, the 3MWT developed in this study is a safe and practical submaximal exercise protocol for healthy adults to predict VO_2_max accurately, even compared to the well-established submaximal exercise protocols, and merits further investigation.

## 1. Introduction

Cardiorespiratory fitness (CRF) is an important indicator of health, various chronic diseases and mortality, which has established itself alongside conventional health indicators such as BMI, blood pressure, drinking and smoking [1,2,3,4]. However, despite its importance and practical implications, the CRF is rarely used in healthcare practice [5,6] due to the fact that the gold standard measure of CRF, maximal oxygen consumption (VO_2_max), requires expensive equipment, trained specialists and maximal effort by participants until volitional fatigue [7]. Due to health risks, cost and inconvenience of the VO_2_max test, submaximal exercise protocols, which do not use a metabolic cart, have been developed to estimate one’s CRF [8,9,10,11,12,13]. Indirect submaximal exercise protocols generally require less expensive specialized equipment, are easier and safer to perform and are utilized more frequently in clinical practice and epidemiological studies. The main variables used in the equations to predict VO_2_max include HR (HR) variables. One of the HR variables commonly utilized is the resting heart rate (RHR), an important vital sign and indicator of CRF. Elevated RHR shows associations with various health conditions including cardiovascular and non-cardiovascular mortality [14,15,16]. HR during exercise is also a common indicator that reflects the intensity of exercise. Previous submaximal protocols with set or standardized workloads have, therefore, used HR to extrapolate or predict VO_2_max [12,13,17]. Finally, HR recovery after submaximal exercise has also been used to predict VO_2_max. A delayed HR recovery is a recognized risk factor for cardiovascular events and mortality [18,19].

Recent research studies on VO_2_max prediction have incorporated the use of technology and have gone as far as estimating VO_2_max without the need to perform predefined protocols. Cao et al. used objectively measured physical activity levels to predict VO_2_max, and Altini et al. used wearable sensors to identify context-specific intensity during ‘free-living’ and used HR to predict VO_2_max [20,21]. Although such approaches are novel and offer easy means to predict VO_2_max, these methods are still being tested and require specialized equipment, making it difficult for the general population to use. When these prediction methods are validated and become more readily available, the knowledge and testing of VO_2_max values may become commonplace. However, despite its potential health implications, the current understanding of VO_2_max in the general population is lacking [6].

This discrepancy may partially be caused by the limitations of the previously developed submaximal exercise protocols such as being complicated or inconvenient to perform, being too strenuous or requiring access to specialized equipment or trained specialists. Additionally, the majority of submaximal protocols used today were developed several decades ago and do not utilize the technology which has become readily available today. Hence, there is still a need to develop a submaximal exercise protocol that is safe, easy and convenient to perform by using tools that are readily available to the general public. Therefore, the purpose of this study was to test the feasibility of simple 3 min walk test (3MWT) protocols in healthy adults and to test if HR responses during the 3MWT protocols can be used to estimate VO_2_max in healthy adults.

## 2. Method

### 2.1. Participants

A total of 60 adults (40 males and 20 females) were included in this study. Participants between the ages of 18 and 40 years were recruited by using convenience sampling, and all participants were screened for contraindications of exercise using a physical activity readiness questionnaire (PAR-Q) [22] before testing. The experimental protocol was approved by the Institutional Review Board of Yonsei University, and all methods were carried out in accordance with the Declaration of Helsinki. All participants provided signed informed consent forms.

### 2.2. Procedure

Participants were instructed to abstain from activities that may influence testing such as moderate-high intensity exercise or alcohol consumption 48 h prior to testing and to abstain from ingestion of caffeine, alcohol, energy drinks and heavy meals 3 h prior to testing [23]. On the day of testing, participants were given a detailed explanation of the testing procedures, and the participants were familiarized with all testing protocol and equipment in order to minimize external physical or psychological influence. They were then asked to sign an informed consent form and given a brief questionnaire, including the Korean Global Physical Activity Questionnaire [24]. Participants were provided with light exercise apparel (t-shirt and shorts) for testing. The testing environment was controlled to 24–26 °C before participants arrived. Participants first underwent anthropometric measurements, followed by the 3MWTs and ended with a maximal graded exercise test with direct VO_2_max measurements. The female participants were scheduled for testing on a day that did not coincide with menstruation in order to minimize discomfort and influence on HR [25].

### 2.3. Measurements

Height, weight and body composition were measured by using a stadiometer and Inbody 720 (Biospace Co., Seoul, Korea). The waist circumference and leg length were measured with a standard tape measure, standing with heels 10 cm apart and feet pointing forward. Waist circumference was measured at the naval and leg length was measured from the greater trochanter to the floor on both legs. All measurements were conducted two times, measured to the nearest 0.1 cm and repeated a third time if the previous measurements had a difference greater than 0.5 cm.

### 2.4. The 3 Min Walk Test Protocol

In order to develop a new submaximal walk test, a pretest was conducted to determine the most appropriate cadence. Appropriateness was determined by whether it could be performed safely, whether the diverse range of participants was physically able to perform the cadence and whether the cadence elicited a sufficient exercise response (50–80% predicted HR max). The cadences of 120 steps per minute (spm) and 130 spm were determined to be the most appropriate potential cadences for the walk test out of the 6 cadences tested (100 spm, 110 spm, 120 spm, 130 spm, 140 spm and 150 spm). A walking duration of three minutes was chosen as the duration of exercise to minimize the testing time while ensuring that a steady HR (plateau) was achieved [26]. The final 3MWT protocols involved continuous brisk walking for three minutes followed by two minutes of rest in a standing position. This was performed twice, once at a cadence of 120 spm (3MWT_120_) and once at a cadence of 130 spm (3MWT_130_).

The participants were asked to put on a commercially available Polar H7 (Polar Electro, Finland) chest strap. Continuous HR data from the H7 was saved on a smart phone in real time and was analyzed on a computer. Once the H7 sensor was strapped on and working properly, the participants sat on a chair at the start line and were given detailed instructions regarding the 3MWT. The instructions and verbal encouragements that were given prior to and during the protocol were standardized and rehearsed before testing.

Once the participants reach a resting state, defined as a HR change of ≤3 bpm for 1 min, the participants began the 3MWT. Participants walked back and forth along a 30 m course marked out on the ground, covering as much ground as possible at a cadence of 120 spm for three minutes (3MWT_120_), followed by two minutes of rest. The steps were walked in beat to the tempo from a free metronome app on a smart phone (Tempo version 3). During rest, the metronome was turned off, and the participants were instructed to concentrate on recovery and to minimize activity. The rate of perceived exertion (RPE) was measured at the end of rest, using a 15-point Borg RPE scale, and the total distance covered was recorded. This protocol was repeated at a faster cadence of 130 spm (3MWT_130_) once the participants achieved a resting state. Participants were instructed to walk in beat to the metronome, and participants that missed ≥6 steps (95% of protocol) for either 3MWT protocols were excluded from the analysis.

### 2.5. HR Variables

Continuous HR data was collected and stored in real time using the Heart Rate Variability Logger (by Marco Altini) App. For analysis, HR was then calculated from the RR intervals provided on the App. HR variables included RHR; HR during exercise at 1 min (HR_1m_), 2 min (HR_2m_) and 3 min (HR_3m_); and HR recovery at 30 s (HRR_30s_), 1 min (HRR_1m_) and 2 min (HRR_2m_). Furthermore, additional HR variables were calculated by using measured HR variables in an effort to increase the validity of the VO_2_max prediction equations. Advancements in technology have now made it possible to measure continuous HR with ease (before, during and after exercise) and to have the data analyzed automatically. Calculated variables in this study are categorized into four categories: (1) dynamics of HR response to walking or how quickly the HR increases once walking begins, i.e., RHR to HR at 1, 2 and 3 min of exercise (HR_1m_–RHR, HR_2m_–RHR, HR_3m_–RHR, HR_2m_-HR_1m_, HR_3m_–HR_2m_, respectively); (2) recovery or the decrease in heart rate after cessation of walking, i.e., HR from exercise at 3 min to 30 s, 1 min and 2 min of rest (HRR_30s_–HR_3m_, HRR_1m_–HR_3m_, HRR_2m_–HR_3m_, HRR_30s_–HRR_1m_, HRR_30s_–HRR_2m_, HRR_1m_–HRR_2m_, respectively); (3) total change in HR or HR response (square root ((increase in HR during exercise)^2^ + (decrease in HR after exercise)^2^))(RHR to HR_3m_ to HRR_30s_, RHR to HR_3m_ to HRR_1m_, RHR to HR_3m_ to HRR_2m_); (4) ability to recover after exercise or the difference in HR between RHR and HR recovery at 30 s, 1 min and 2 min (HRR_30s_–RHR, HRR_1m_–RHR, HRR_2m_–RHR, respectively).

### 2.6. Maximal Graded Exercise Protocol

The Bruce protocol was used to directly measure VO_2_max and was conducted in accordance to the standard procedure [27]. The Q-Stress TM65 treadmill (Cardiac Science Co., WA, USA) was used for testing, and the MMS-2400 metabolic cart (Cardiac Science Co., USA), which is compatible with the Polar H7, was used to collect data. All instructions regarding the protocol and safety, as well as verbal encouragements, were standardized and rehearsed before testing. The lead investigator monitored the data during testing while an additional investigator monitored the participants during testing to ensure safety. The protocol continued until volitional fatigue, at which point the treadmill gradient was immediately lowered to 0° and the speed reduced to a slow walking pace for five minutes. In order for the results to be considered a valid VO_2_max, two of the following three criteria needed to be met: respiratory exchange ratio of >1.1, maximal HR of >85% of age predicted maximal HR and an RPE ≥18 on the Borg scale.

### 2.7. Statistical Analysis

All analyses were performed using SPSS 22 (IBM, NY, USA) with a level of significance set at *p* < 0.05. Differences between means in the descriptive analysis were compared by using analysis of variance (ANOVA). The Pearson correlation analysis was conducted to identify any possible independent variables that were then included in the final prediction equation. After possible independent variables were identified, stepwise regression analysis was used to determine which variables best predicted VO_2_max. Four sets of equations were developed using the following: (1) no HR variables, (2) only HR variables that were directly measured, (3) only HR variables that were calculated using two or more measured HR variables and (4) a combination of both measured and calculated HR variables.

## 3. Results

### 3.1. Participant Characteristics

A total of 60 participants were included for analysis, of which 40 were males and 20 were females. Participant characteristics are presented in Table 1. The mean age of both the male and female participants was 26 years of age. The majority of participants had ‘good’ or ‘very good’ VO_2_max scores when compared to Korean sex and age-appropriate reference values.

### 3.2. HR Characteristics

**Figure 1:**
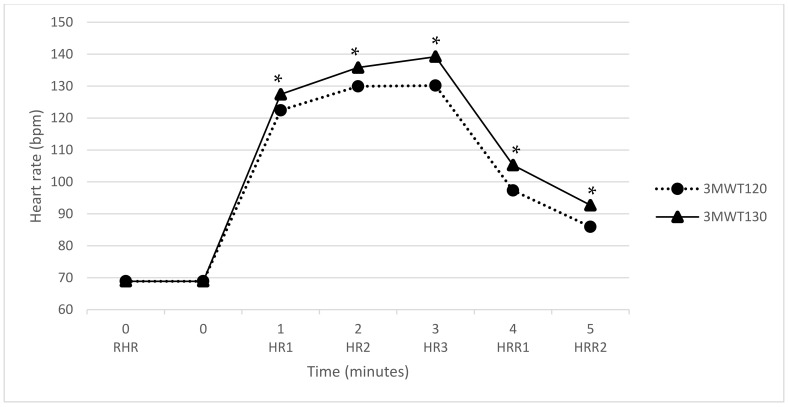
Heart rate response during the 3MWT at a cadence of 120 spm and 130 spm; * significant difference of *p* < 0.05. Abbreviation: 3MWT_120_, 3 min walk test at a cadence of 120 spm; 3MWT_130_, 3 min walk test at a cadence of 130 spm; spm, steps per minute; RHR, resting heart rate; HR1, heart rate at 1 min of exercise; HR2, heart rate at 2 min of exercise; HR3, heart rate at 3 min of exercise; HRR1, heart rate recovery at 1 min; HRR2, heart rate recovery at 2 min.

The mean HR responses to the 3MWT_120_ and the 3MWT_130_ can be observed in Figure 1. The mean HR was 69 bpm at rest, reached 130 bpm and 139 bpm during exercise and dropped to 86 bpm and 93 bpm after two minutes of recovery for the 3MWT_120_ and the 3MWT_130_, respectively.

HR variables during and after exercise were significantly higher in the 3MWT_130_ compared to the 3MWT_120_. HR responses according to sex for the 3MWT_120_ and the 3MWT_130_ are presented in Table 2. There were significant differences in HR responses to the 3MWT between males and females, with females showing higher HRs during the 3MWT. There were also significant differences between HR responses to the 3MWT_120_ and the 3MWT_130_, with HR being significantly higher during walking and recovery for 3MWT_130_ in both men and women. The total distance covered and RPE scores were also significantly higher for the 3MWT_130_.

Correlations between anthropometric variables and HR during the 3MWT are presented in Table 3. The VO_2_max values were significantly correlated with sex, height, weight, leg length and total distance walked. The total distance walked and RPE scores during the 3MWT were significantly associated with all HR variables during the 3MWT at two different cadences and recovery. Although not presented in the table, total distance walked was also significantly correlated with sex, height, weight and leg length.

Correlations of measured and calculated HR variables with VO_2_max are presented in Table 4. For both the 3MWT_120_ and the 3MWT_130_, most measured HR variables showed weak but significant correlations with VO_2_max. On the other hand, most calculated HR variables showed weaker correlations with VO_2_max and did not show significant correlations with VO_2_max.

Table 5 shows the equations used to predict measured VO_2_max values using no HR variables, only measured HR variables, only calculated HR variables or a combination of both measured and calculated HR related variables. Equations that used no HR variables showed the lowest r-values (3MWT: r = 0.75) and r^2^-values (3MWT: r^2^ = 0.57). Equations that used a combination of both measured and calculated HR related variables resulted in the highest r-values (3MWT_120_: r = 0.84; 3MWT_130_: r = 0.81) and r^2^-values (3MWT_120_: r^2^ = 0.70; 3MWT_130_: r^2^ = 0.65), while the 3MWT_120_ also had the smallest SEE (3MWT_120_: SEE = 4.80 mL/kg/min).

## 4. Discussion

The data of 60 participants was used to test the feasibility of a new submaximal walk test developed in this study to predict VO_2_max. Correlation analysis showed VO_2_max to be significantly associated with anthropometric variables and measured HR related variables but less so with the calculated HR related variables. Multiple regression analyses were conducted to develop four equations for both the 3MWT_120_ and the 3MWT_130_ that used no HR variables, only measured HR variables, only calculated HR variables and a combination of both measured and calculated HR related variables. The set of equations that used a combination of both measured and calculated HR related variables best predicted measured VO_2_max values. All equations only included variables that can be measured using basic tools and a HR monitor. Although total distance covered during the 3MWT and RPE showed a significant correlation with VO_2_max, these variables did not increase the accuracy of the predictions and were excluded from the final equation, thereby making the 3MWT more practical and convenient to conduct.

The diverse range of calculated HR related variables was investigated to identify new potential HR variables that increased the accuracy of the prediction equation. Calculated HR related variables showed weak correlations with VO_2_max values that failed to reach statistical significance. Although no new HR related variables were identified, equations that used a combination of both measured and calculated HR related variables showed improved accuracy in predicting measured VO_2_max values. For the final equation, the calculated HR variables were squared to exaggerate the differences. It was surprising to find that the final prediction equation using a combination of measured and calculated HR variables for the 3MWT_120_ was better at estimating VO_2_max than the equation for the 3MWT_130_, since the 3MWT_130_ elicited a higher exercise response. This suggests that the 3MWT_120_ may be the more feasible protocol of the two that were tested. However, a larger, more representative sample of the general population is needed before the most appropriate 3MWT can be determined.

A limitation of this study is the relatively small sample size of young healthy adults, 56.7% of which were classified as fit according to the Korean sex and age-appropriate reference values. Although this sample may not be representative of the general population, a protocol that can be used to accurately predict VO_2_max in the general population would require the protocol to elicit a sufficient exercise response in a young fit population while being easy enough for the aged unfit population to perform. In this regard, the sample of this pilot test is the most appropriate sample for investigating the feasibility of this protocol before testing on an older, less healthy sample. A study that used a similar sample size (*n* = 53 and *n* = 27) of fit college-aged participants for predicting VO_2_max by using the one-mile run and 1.5 mile run found r-values of 0.87 and 0.90, respectively [13]. Another study that used a small homogenous sample (38 females) to compare the ability of commonly used submaximal exercise protocols to predict VO_2_max found r-values of r = 0.73, r = 0.79, r = 0.55 and r = 0.66 for the 1.5 mile run, the one-mile walk test, the Queen’s step test and the Astrand–Rhyming nomogram using the extrapolation method, respectively [28]. Another study that used a small homogenous sample (21 young well-trained men) found the American College of Sports Medicine’s submaximal treadmill running test and a modified version of the same test to predict VO_2_max and found r-values of r = 0.65 and r = 0.64, respectively [29]. Although the findings of the current study cannot be directly compared to the findings of previous studies that have investigated the ability of submaximal exercise protocols to predict VO_2_max on small homogenous samples, they do provide a reference for the findings of this study. The results of the 3MWT are encouraging and warrant further examination, especially since the r-values fared well compared to the results of studies that investigated the most commonly used submaximal protocols. Compared to ‘gold-standard’ submaximal protocols, the 3MWT also requires less specialized equipment, less strenuous activity and minimum supervision while being more convenient to perform. This study used a gold standard HR monitor to measure continuous HR because, although studies have validated the use of smart phones to monitor HR, further investigations are needed to understand the influence of different smartphone features before a consensus on its use in healthcare can be established [30]. However, if the use of smartphones as HR monitors were to become common practice in healthcare, the 3MWT would provide a convenient method for estimating VO_2_max for anyone owning a smartphone.

In conclusion, this study was able to develop a safe, convenient and more practical submaximal exercise protocol that is able to predict VO_2_max accurately (r = 0.84, r^2^ = 0.70, SEE = 4.80 mL/kg/min). This study also identified new HR related variables that can be used to increase the accuracy of VO_2_max prediction, while using basic tools that are easily accessible. Although the small homogenous sample makes it difficult to generalize, the initial results are promising and warrant further study in a larger and more heterogeneous population.

## Figures and Tables

**Table 1:** Participants’ physical characteristics.

Variables	Total (*n* = 60)	Male (*n* = 40)	Female (*n* = 20)
Age (years)	26.35 ± 4.52	26.50 ± 4.25	26.05 ± 5.13
Height (cm)	173.50 ± 8.29	177.79 ± 5.58	164.92 ± 5.76 *
Weight (kg)	71.67 ± 12.20	77.99 ± 9.20	59.03 ± 6.03 *
Body Mass Index (kg/m^2^)	23.68 ± 2.84	26.69 ± 2.75	21.68 ± 1.78 *
Waist Circumference (cm)	80.64 ± 7.92	93.45 ± 7.45	85.02 ± 5.56 *
Leg Length (cm)	86.61 ± 4.64	88.72 ± 3.56	82.38 ± 3.54 *
Body Fat Percent (%)	20.86 ± 6.10	17.95 ± 5.30	26.68 ± 4.02 *
Body Muscle Percent (%)	44.59 ± 6.35	46.86 ± 3.19	40.05 ± 2.31 *
Maximal Oxygen Consumption (ml/kg/min)	44.98 ± 7.24	48.33 ± 5.96	38.29 ± 4.37 *
Fit individuals ^a^ (%)	56.7%	60%	50%

Data is presented as mean ± standard deviation; * significant difference of *p* < 0.05, between males and females; ^a^ maximal oxygen consumption results were compared to the Korean age and sex appropriate reference values for males and females, and those with ‘good’ or ‘very good’ were classified as ‘fit individual’s.

**Table 2:** Characteristics of the measured exercise related variables.

Variables		Male (*n* = 40)	Female (*n* = 20)
Resting Heart Rate		68.18 ± 9.74	70.25 ± 8.00
3MWT at a cadence of 120 spm			
	HR_1m_ (bpm)	119.15 ± 12.98	128.90 ± 11.24 *
	HR_2m_ (bpm)	125.15 ± 15.61	139.45 ± 14.65 *
	HR_3m_ (bpm)	126.78 ± 15.89	139.50 ± 17.32 *
	HRR_30s_ (bpm)	109.08 ± 15.99	120.35 ± 22.96 *
	HRR_1m_ (bpm)	94.73 ± 14.53	100.85 ± 19.03
	HRR_2m_ (bpm)	85.38 ± 14.03	87.05 ± 15.17
	Distance walked (m)	346.29 ± 25.09	331.40 ± 20.26 *
	RPE	10.90 ± 2.71	11.75 ± 1.59
3MWT at a cadence of 130 spm			
	HR_1m_ (bpm)	124.50 ± 14.28 ^#^	133.30 ± 14.25 *^#^
	HR_2m_ (bpm)	132.48 ± 17.60 ^#^	143.70 ± 16.82 *^#^
	HR_3m_ (bpm)	134.78 ± 19.06 ^#^	148.05 ± 16.58 *^#^
	HRR_30s_ (bpm)	119.65 ± 17.53 ^#^	130.20 ± 20.52 *^#^
	HRR_1m_ (bpm)	103.78 ± 15.88 ^#^	108.60 ± 20.49 ^#^
	HRR_2m_ (bpm)	91.13 ± 14.12 ^#^	95.70 ± 17.00 ^#^
	Distance walked (m)	368.85 ± 23.79 ^#^	350.38 ± 19.06 *^#^
	RPE	12.08 ± 1.99 ^#^	12.30 ± 1.75 ^#^

Data is presented as mean ± standard deviation; * significant difference of *p* < 0.05, between males and females; ^#^ significant difference of *p* < 0.05, between 3MWT at a cadence of 120 spm and 130 spm. Abbreviations: VO_2_max, maximal oxygen consumption; 3MWT, 3 min walk test; spm, steps per minute; HR_1m_, heart rate at 1 min; HR_2m_, heart rate at 2 min; HR_3m_, heart rate at 3 min; HRR_30s_, heart rate recovery at 30 s; HRR_1m_, heart rate recovery at 1 min; HRR_2m_, heart rate recovery at 2 min; RPE, rate of perceived exertion.

**Table 3:** Correlation of anthropometric measurements and exercise related variables.

		Sex	Age	Height	Weight	BMI	WC	LL	Dist	RPE
VO_2_max (mL/kg/min)		−0.66 **	−0.20	0.39 **	0.39 **	0.25	0.14	0.47 **	0.28 *	−0.20
RHR		0.11	−0.15	0.03	−0.01	−0.04	0.01	−0.02	−0.08	0.26 *
3MWT at a cadence of 120 spm										
	HR_1m_ (bpm)	0.35 **	−0.22	−0.16	−0.26 *	−0.25	−0.17	−0.16	0.31 *	0.39 **
	HR_2m_ (bpm)	0.41 **	−0.11	−0.20	−0.31 *	−0.28 *	−0.18	−0.22	0.32 *	0.37 **
	HR_3m_ (bpm)	0.30 **	−0.12	−0.14	−0.25	−0.25	−0.15	−0.18	0.39 **	0.38 **
	HRR_30s_ (bpm)	0.28 *	−0.10	−0.11	−0.25	−0.26	−0.21	−0.18	0.38 **	0.41 **
	HRR_1m_ (bpm)	0.21	−0.11	−0.05	−0.20	−0.24	−0.15	−0.11	0.31 *	0.38 **
	HRR_2m_ (bpm)	0.06	−0.11	−0.17	−0.17	−0.27 *	−0.17	−0.03	0.26 *	0.31 *
3MWT at a cadence of 130 spm										
	HR_1m_ (bpm)	0.28 *	−0.31 *	−0.24	−0.24	−0.27 *	−0.17	−0.10	0.39 **	0.36 **
	HR_2m_ (bpm)	0.27 *	−0.13	−0.18	−0.17	−0.18	−0.07	−0.10	0.43 **	0.40 **
	HR_3m_ (bpm)	0.33 *	−0.17	−0.26 *	−0.26 *	−0.28 *	−0.13	−0.16	0.41 **	0.50 **
	HRR_30s_ (bpm)	0.27 *	−0.06	−0.23	−0.23	−0.27 *	−0.16	−0.14	0.46 **	0.51 **
	HRR_1m_ (bpm)	0.14	−0.08	−0.15	−0.15	−0.22	−0.11	−0.08	0.42 **	0.48 **
	HRR_2m_ (bpm)	0.14	−0.17	−0.15	−0.15	−0.23	−0.12	−0.05	0.33 *	0.43 **

* Significant correlation of *p* < 0.05 (2-tailed); ** significant correlation of *p* < 0.01 (2-tailed). Abbreviation: BMI, body mass index; WC, waist circumference; LL, leg length; Dist, total distance walked; RPE, rate of perceived exertion; VO_2_max, maximal oxygen consumption; RHR, resting heart rate; 3MWT, 3 min walk test; spm, steps per minute; HR_1m_, heart rate at 1 min; HR_2m_, heart rate at 2 min; HR_3m_, heart rate at 3 min; HRR_30s_, heart rate recovery at 30 s; HRR_1m_, heart rate recovery at 1 min; HRR_2m_, heart rate recovery at 2 min.

**Table 4:** Correlation of maximal oxygen consumption and exercise related variables.

		Correlation between 3MWT_120_ and VO_2_max	Correlation between 3MWT_130_ and VO_2_max
Measured variables			
	RHR (bpm)	−0.27 *	−0.27 *
	HR_1m_ (bpm)	−0.25	−0.19
	HR_2m_ (bpm)	−0.33 *	−0.27 *
	HR_3m_ (bpm)	−0.31 *	−0.29 *
	HRR_30s_ (bpm)	−0.31 *	−0.31 *
	HRR_1m_ (bpm)	−0.26 *	−0.28 *
	HRR_2m_ (bpm)	−0.14	−0.27 *
	Total distance walked (m)	0.22	0.28 *
	RPE	−0.19	−0.20
How fast HR increases with walking			
	HR_1m_-RHR	−0.06	−0.02
	HR_2m_-RHR	−0.18	−0.14
	HR_3m_-RHR	−0.17	−0.16
	HR_2m_-HR_1m_	−0.26 *	−0.29 *
	HR_3m_-HR_2m_	0.03	−0.13
How fast HR decrease once stop walking			
	HR_3m_-HR_1m_	−0.21	−0.31 *
	HRR_30s_-HR_3m_	−0.06	−0.05
	HRR_1m_-HR_3m_	0.12	0.05
	HRR_2m_-HR_3m_	0.24	0.12
	HRR_30s_-HRR_1m_	0.23	0.12
	HRR_30s_-HRR_2m_	0.31 *	0.19
	HRR_1m_-HRR_2m_	0.26 *	0.15
Total change in HR			
	RHR to HR_3m_ to HRR_30s_	−0.10	−0.08
	RHR to HR_3m_ to HRR_1m_	−0.16	−0.11
	RHR to HR_3m_ to HRR_2m_	−0.19	−0.11
How close HR return to RHR			
	HRR_30s_-RHR	−0.20	−0.19
	HRR_1m_-RHR	−0.13	−0.17
	HRR_2m_-RHR	0.05	−0.12

* Significant correlation of *p* < 0.05 (2-tailed). Abbreviation: 3MWT_120_, 3 min walk test at a cadence of 120 spm; 3MWT_130_, 3 min walk test at a cadence of 130 spm; spm, steps per minute; VO_2_max, maximal oxygen consumption; RHR, resting heart rate; HR_1m_, heart rate at 1 mi; HR_2m_, heart rate at 2 min, HR_3m_, heart rate at 3 min; HRR_30s_, heart rate recovery after 30 s of rest; HRR_1m_, heart rate recovery after 1 min of rest; HRR_2m_, heart rate recovery after 2 min of rest; RPE, rate of perceived exertion; RHR to HR_3m_ to HRR_30s_, total change in heart rate from resting heart rate until 30 s after rest; RHR to HR_3m_ to HRR_1m_, total change in heart rate from resting heart rate until 1 min after rest; RHR to HR_3m_ to HRR_2m_, total change in heart rate from resting heart rate until 2 min after rest.

**Table 5:** Maximal oxygen consumption prediction equations.

No Heart Rate Variables			r	r^2^	SEE
	3MWT	−14.62_x1_ − 0.30_x2_ − 1.65_x3_ + 1.89_x4_ − 6.34_x5_ + 372.84	0.75 **	0.57	4.98
Measured heart rate related variables only					
	3MWT_120_	−13.77_x1_ − 0.36_x2_ − 1.56_x3_ + 1.83_x4_ − 6.12_x5_ − 0.14_x6_ + 0.003_x7_ + 0.04_x8−_ + 0.05_x9_ − 0.04_x10_ − 0.13_x11_ + 0.08_x12_ + 364.21	0.79 **	0.63	4.97
	3MWT_130_	−13.98_x1_ − 0.27_x2_ − 1.46_x3_ + 1.72_x4_ − 5.77_x5_ − 0.12_x6_ + 0.11 _x13_ − 0.10_x14_ + 0.12_x15_ − 0.03_x16_ − 0.12_x17_ + 0.02_x18_ + 343.86	0.80 **	0.65	4.94
Calculated heart rate related variables only					
	3MWT_120_	−14.92_x1_ − 0.35_x2_ − 1.46_x3_ + 1.62_x4_ − 5.39_x5_ − 0.12_x6_ − 0.33_x19_ −0.30_x20_ − 0.09_x21_ + 0.32_x22_ + 0.18_x23_ + 0.10_x24_ + 0.11_x25_ + 0.62 _x26_ + 342.72	0.81 **	0.66	4.87
	3MWT_130_	−13.71_x1_ − 0.28_x2_ − 1.42_x3_ + 1.67_x4_ − 5.66_x5_ − 0.146_x6_ − 0.06_x27_ −0.16_x28_ − 0.02_x29_ − 0.09_x30_ − 0.40_x31_ + 0.32_x32_ + 0.46_x33_ − 0.45 _x34_ + 337.86	0.80 **	0.64	5.02
Combination of measured and calculated heart rate variables					
	3MWT_120_	−16.05_x1_ − 0.36_x2_ − 1.55_x3_ + 1.72_x4_ − 5.90_x5_ − 0.18_x6_ + 0.21_x7_ + 0.01_x8_ − 0.13_x9_ − 0.25_x11_ + 0.27_x12_ − 0.001_x35_ + 0.002_x36_ + 0.04_x37_ − 0.001_x38_ + 0.04_x39_ + 0.001_x40_ + 0.003_x41_ − 0.003_x42_ + 361.49	0.84 **	0.70	4.80
	3MWT_130_	−13.13_x1_ − 0.34_x2_ − 1.43_x3_ + 1.75_x4_ − 5.82_x5_ + 0.11_x6_ + 0.01 _x13_ − 0.29_x14_ + 0.60_x15_ − 0.62_x17_ + 0.52_x18_ + 0.003_x43_ + 0.01_x44_ + 0.01_x45_ − 0.01_x46_ + 0.01_x47_ + 0.001_x48_ + 0.001_x49_ − 0.001_x50_ + 339.94	0.81 **	0.65	5.25

** Significance of *p* < 0.001. SEE presented as mL/kg/min. Abbreviation: SEE, standard error of the estimate; 3MWT_120_, 3 min walk test at a cadence of 120 spm; 3MWT_130_, 3 min walk test at a cadence of 130 spm; spm, steps per minute; _x1_, sex; _x2_, age; **_x3_**, height; **_x4_**, weight; **_x5_**, body mass index; **_x6_**, resting heart rate; **_x7_**, heart rate(_120_) at 1 min; **_x8_**, heart rate(_120_) at 2 min; **_x9_**, heart rate(_120_) at 3 min; **_x10_**, heart rate recovery(_120_) at 30 s; **_x11_**, heart rate recovery(_120_) at 1 min; **_x12_**, heart rate recovery(_120_) at 2 min; **_x13_**, heart rate(_130_) at 1 min; **_x14_**, heart rate(_130_) at 2 min; **_x15_**, heart rate(_130_) at 3 min; **_x16_**, heart rate recovery(_130_) at 30 s; **_x17_**, heart rate recovery(_130_) at 1 min; **_x18_**, heart rate recovery(_130_) at 2 min; **_x19_**, difference between resting heart rate and heart rate(_120_) at 1 min; **_x20_**, difference between heart rate(_120_) at 1 min and 2 min; **_x21_**, difference between heart rate(_120_) at 2 min and 3 min; **_x22_**, difference between heart rate(_120_) at 3 min and heart rate recovery(_120_) at 1 min; **_x23_**, difference between heart rate recovery(_120_) at 1 min and heart rate recovery(_120_) at 2 min; **_x24_**, total change in heart rate from resting heart rate until 30 s after rest (_120_); **_x25_**, total change in heart rate from resting heart rate until 1 min after rest (_120_); **_x26_**, total change in heart rate from resting heart rate until 2 min after rest (_120_); **_x27_**, difference between resting heart rate and heart rate(_130_) at 1 min; **_x28_**, difference between heart rate(_130_) at 1 min and 2 min; **_x29_**, difference between heart rate(_130_) at 2 min and 3 min; **_x30_**, difference between heart rate(_130_) at 3 min and heart rate recovery(_130_) at 1 min; **_x31_**, difference between heart rate recovery(_130_) at 1 min and heart rate recovery(_130_) at 2 min; **_x32_**, total change in heart rate from resting heart rate until 30 s after rest (_130_); **_x33_**, total change in heart rate from resting heart rate until 1 min after rest (_130_); **_x34_**, total change in heart rate from resting heart rate until 2 min after rest (_130_); **_x35_**, squared difference between resting heart rate and heart rate(_120_) at 1 min; _**x36**_, squared difference between heart rate(_120_) at 1 min and 2 min; **_x37_**, squared difference between heart rate(_120_) at 2 min and 3 min; **_x38_**, squared difference between heart rate (_120_) at 3 min and heart rate recovery(_120_) at 1 min; **_x39_**, squared difference between heart rate recovery(_120_) at 1 min and heart rate recovery(_120_) at 2 min; _**x40**_, squared total change in heart rate from resting heart rate until 30 s after rest (_120_); _**x41**_, squared total change in heart rate from resting heart rate until 1 min after rest (_120_); _**x42**_, squared total change in heart rate from resting heart rate until 2 min after rest (_120_); _**x43**_, squared difference between resting heart rate and heart rate(_130_) at 1 min; **_x44_**, squared difference between heart rate(_130_) at 1 min and 2 min; **_x45_**, squared difference between heart rate (_130_) at 2 min and 3 min; **_x46_**, squared difference between heart rate(_130_) at 3 min and heart rate recovery(_130_) at 1 min; **_x47_**, squared difference between heart rate recovery(_130_) at 1 min and heart rate recovery(_130_) at 2 min; _**x48**_, squared total change in heart rate from resting heart rate until 30 s after rest (_130_); _**x49**_, squared total change in heart rate from resting heart rate until 1 min after rest (_130_); _**x50**_, squared total change in heart rate from resting heart rate until 2 min after rest (_130_).

## Data Availability

The datasets generated and/or analyzed during the current study are available from the corresponding author upon reasonable request.
